# Ileal microbial shifts after Roux-en-Y gastric bypass orchestrate changes in glucose metabolism through modulation of bile acids and L-cell adaptation

**DOI:** 10.1038/s41598-021-03396-4

**Published:** 2021-12-10

**Authors:** Jerry T. Dang, Valentin Mocanu, Heekuk Park, Michael Laffin, Caroline Tran, Naomi Hotte, Shahzeer Karmali, Daniel W. Birch, Karen Madsen

**Affiliations:** 1 Division of General Surgery, Department of Surgery, University of Alberta Hospital, University of Alberta, 8440 112 Street NW, Edmonton, AB T6G 2B7 Canada; 2 Department of Medicine, Columbia University, New York City, NY USA; 3 Department of Biological Sciences, University of Alberta, Edmonton, AB Canada; 4 Department of Medicine, University of Alberta, Edmonton, AB Canada

**Keywords:** Bacteria, Gastrointestinal hormones, Diabetes, Metabolic syndrome, Obesity

## Abstract

Roux-en-Y gastric bypass (RYGB)-induced glycemic improvement is associated with increases in glucagon-like-peptide-1 (GLP-1) secreted from ileal L-cells. We analyzed changes in ileal bile acids and ileal microbial composition in diet-induced-obesity rats after RYGB or sham surgery to elucidate the early and late effects on L-cells and glucose homeostasis. In early cohorts, there were no significant changes in L-cell density, GLP-1 or glucose tolerance. In late cohorts, RYGB demonstrated less weight regain, improved glucose tolerance, increased L-cell density, and increased villi height. No difference in the expression of GLP-1 genes was observed. There were lower concentrations of ileal bile acids in the late RYGB cohort. Microbial analysis demonstrated decreased alpha diversity in early RYGB cohorts which normalized in the late group. The early RYGB cohorts had higher abundances of *Escherichia–Shigella* but lower abundances of *Lactobacillus*, *Adlercreutzia*, and *Proteus* while the late cohorts demonstrated higher abundances of *Escherichia–Shigella* and lower abundances of *Lactobacillus*. Shifts in *Lactobacillus* and *Escherichia–Shigella* correlated with decreases in multiple conjugated bile acids. In conclusion, RYGB caused a late and substantial increase in L-cell quantity with associated changes in bile acids which correlated to shifts in *Escherichia–Shigella* and *Lactobacillus*. This proliferation of L-cells contributed to improved glucose homeostasis.

## Introduction

Roux-en-Y gastric bypass (RYGB) leads to rapid and sustained resolution of diabetes, however the mechanisms responsible for these dramatic corrections in maladaptive glucose homeostasis remain unclear^1^. Two major theories have been proposed to explain the metabolic changes associated with diabetes remission. First, the hindgut hypothesis postulates that higher concentrations of undigested nutrients reach the distal intestine enhancing the release of hormones such as glucagon-like-peptide-1 (GLP-1). Alternatively, the foregut hypothesis suggests that bypassing the proximal intestine reduces secretion of anti-incretin hormones. Intestinal enteroendocrine L-cells which secrete GLP-1 are thought to be key players underlying these two theories but little is known about how their post-operative adaptations influence metabolic improvement, nor the factors which may influence their translational capacity^2^.

Enteroendocrine L-cells are present throughout the small and large bowel but are greatest in number within the distal ileum. Mechanisms by which RYGB influences L-cell changes that subsequently result in beneficial increases in GLP-1 are not entirely clear but emerging evidence has implicated several factors including surgical-induced changes in the gut microbiome and circulating bile acids. In a study of L-cell expression and gut microbes, Arora et al. found that colonization of germ-free mice with microbes from conventional mice resulted in transcriptional suppression of L-cells^3^. It is plausible that a rapid modulation of the enteric microbiome following RYGB alters gut microbial function with a resultant change in signaling to L-cells. Furthermore, studies consistently demonstrate profound RYGB-mediated changes to serum bile acid concentration and composition thought to occur due to intestinal adaptations that lead to increased intestinal absorption of nutrients^4^. These differences warrant further study as changes to ileal bile acid composition modulate GLP-1 secretion through regulatory bile acid receptors TGR5^5^ and FXR^6^, and in turn also significantly influence gut microbial composition^7–10^.

Ultimately, the complex changes that occur within the gut microbiota and enteric bile acid profiles after RYGB could lead to modulation of gene expression within L-cells with a subsequent increase in GLP-1 production and improvement in glucose homeostasis. We hypothesize that altered gut anatomy and bile acid physiology following RYGB leads to significant changes in gut microbial composition and function resulting in altered gene expression within ileal L-cells and increased release of GLP-1 and that early increases in GLP-1 will be modulated by transcriptional changes within L-cells and later through a proliferation in the quantity of L-cells. The primary objective of this study was to determine early and late changes to L-cell quantity and GLP-1 gene expression of ileal L-cells between RYGB and sham surgery, defined as 2 weeks and 14 weeks, respectively. Secondary objectives included determining changes in villi morphology, bile acid composition, and microbial composition within the ileum and identifying pathways in which these changes affect L-cell gene expression and quantity. Additionally, serum GLP-1 and glucose tolerance were compared between groups to determine the effects of RYGB on glucose metabolism.

## Results

### RYGB reduces body weight gain in rats fed with a high-fat diet

**Figure 1:**
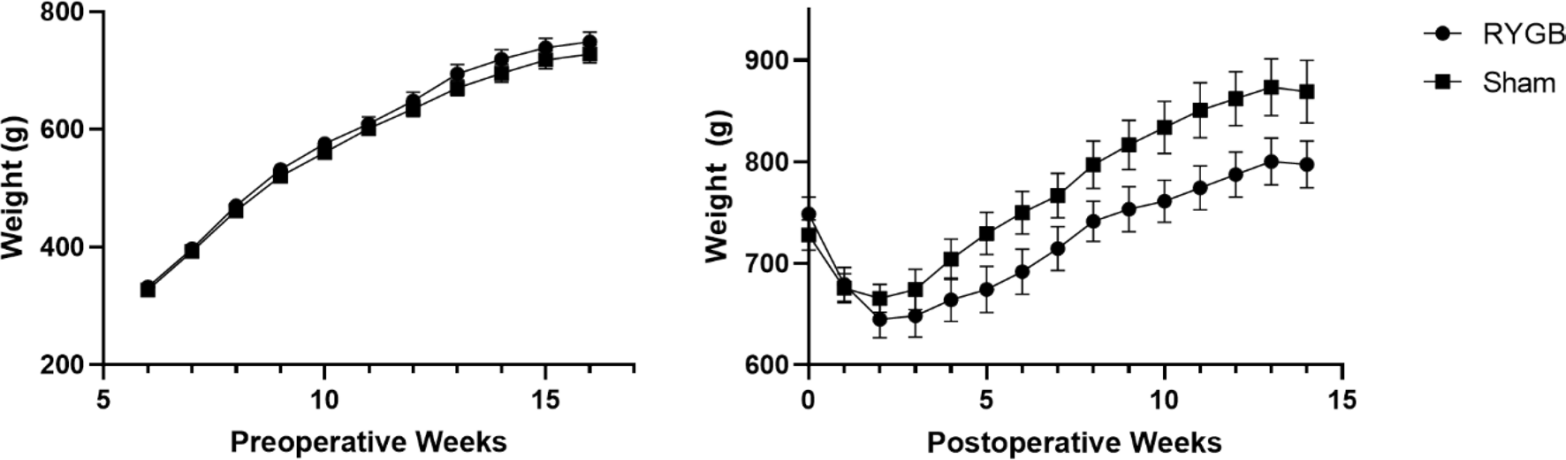
Pre- and post-operative absolute weight on high fat diet; RYGB, Roux-en-Y gastric bypass.

Rats with diet-induced obesity underwent either RYGB or sham surgery. There was significant weight gain from weeks 6 to 16 in both the RYGB and sham cohorts after introduction of a high fat diet (%weight change, 125.1 ± 3.9% vs. 122.3 ± 3.6%, *p* = 0.6, Fig. 1). Following surgery, there was an initial loss of weight in the first 2 weeks due to post-operative dietary restrictions in both RYGB and sham animals. However, rats undergoing RYGB had significantly less weight gain at 14 weeks postoperatively compared to sham (6.4 ± 2.5 vs. 23.7 ± 2.0%, *p* = 0.0001, Fig. 1).

Overall, 88.9% of RYGB rats and 93.8% of sham rats survived to study endpoints. Rats that survived to endpoint did not have any complications and none of the rats demonstrated bile-salt wasting diarrhea. All rats with unexpected mortality underwent necropsy by a veterinarian. Deaths were due to aspiration pneumonitis, fascial dehiscence and anastomotic leak at the gastrojejunostomy.

### RYGB promotes increased ileal villi length, crypt width and crypt depth in the late cohort

**Figure 2:**
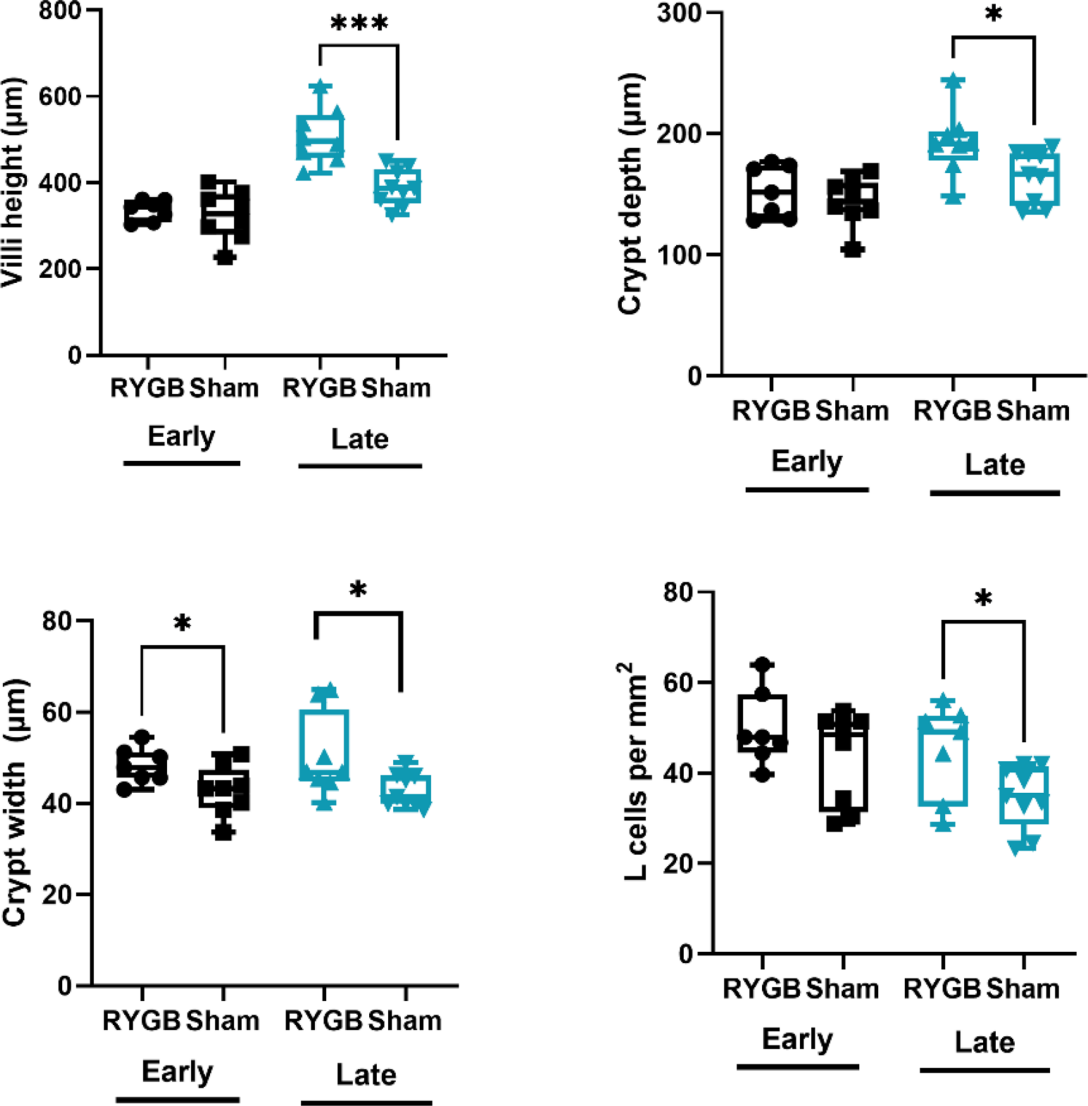
Ileal morphological changes and L-cell density amongst groups: early Roux-en-Y gastric bypass (RYGB) (n = 7), early sham (n = 8), late RYGB (n = 8), late sham (n = 9). Error bars on figures represent standard error of the means and asterisks represent statistical significance with * as *p* < 0.05, ** as *p* < 0.01, *** as *p* < 0.001, **** as *p* < 0.0001.

Early cohort RYGB rats had increases in ileal crypt width versus sham (48.3 ± 3.9 vs. 42.8 ± 5.4 μm, *p* = 0.04). When comparing the late cohorts, RYGB rats experienced additional significant morphological differences including increased villi height (507.7 ± 64.7 vs. 388.8 ± 42.2 μm, *p* = 0.0004), crypt width (50.3 ± 9.1 vs. 43.0 ± 3.7 μm, *p* = 0.04) and crypt depth (192.9 ± 27.0 vs. 165.3 ± 21.4 μm, *p* = 0.03, Fig. 2). Villi width and epithelial thickness were similar amongst all groups.

### RYGB results in increased ileal L-cell density in the late cohort

Immunofluorescence demonstrated multiple L-cells within ileal tissue but only rare occurrences of K- or LK-cells. In the early cohorts, L-cell density was not significantly different but was significantly increased in the late RYGB cohort (45.0 ± 10.5 vs. 34.7 ± 7.0 cells/mm^2^, *p* = 0.03, Fig. 2). Despite increases in L-cell density, there were no significant differences in GLP-1 gene expression within L-cells between cohorts for *gcg* or *PC1/3* at either time point.

### RYGB improves glucose tolerance in the late cohort

**Figure 3:**
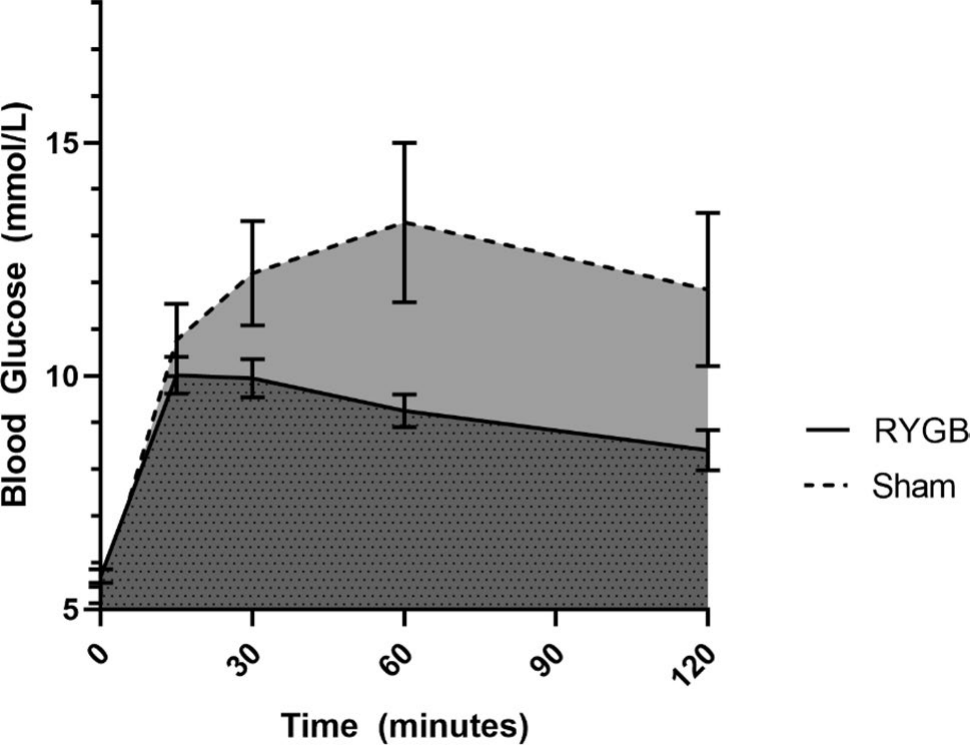
Intraperitoneal glucose tolerance testing in gastric bypass (n = 8) versus sham (n = 9) in the late cohorts; RYGB, Roux-en-Y gastric bypass.

There were no differences in glucose tolerance testing in the early groups. However, in the late groups, dynamic glycemic responses to an intraperitoneal glucose tolerance test revealed a significantly lower area under the curve after RYGB compared to sham (18.1 ± 0.9 vs. 23.8 ± 3.9 mmol-h/L, *p* = 0.046, Fig. 3).

### GLP-1 was higher in the late RYGB cohort

Post-prandial serum GLP-1 was similar between the early RYGB and early sham groups. However, GLP-1 was more than two times greater in the late RYGB compared to the late sham cohort (45.4 ± 48.2 vs. 21.1 ± 6.0 pM, *p* = 0.12) but this did not reach statistical significance. Serum GIP did not differ between groups at either time point.

### RYGB causes an early decrease in ileal microbial diversity which was restored in the late cohort

**Figure 4:**
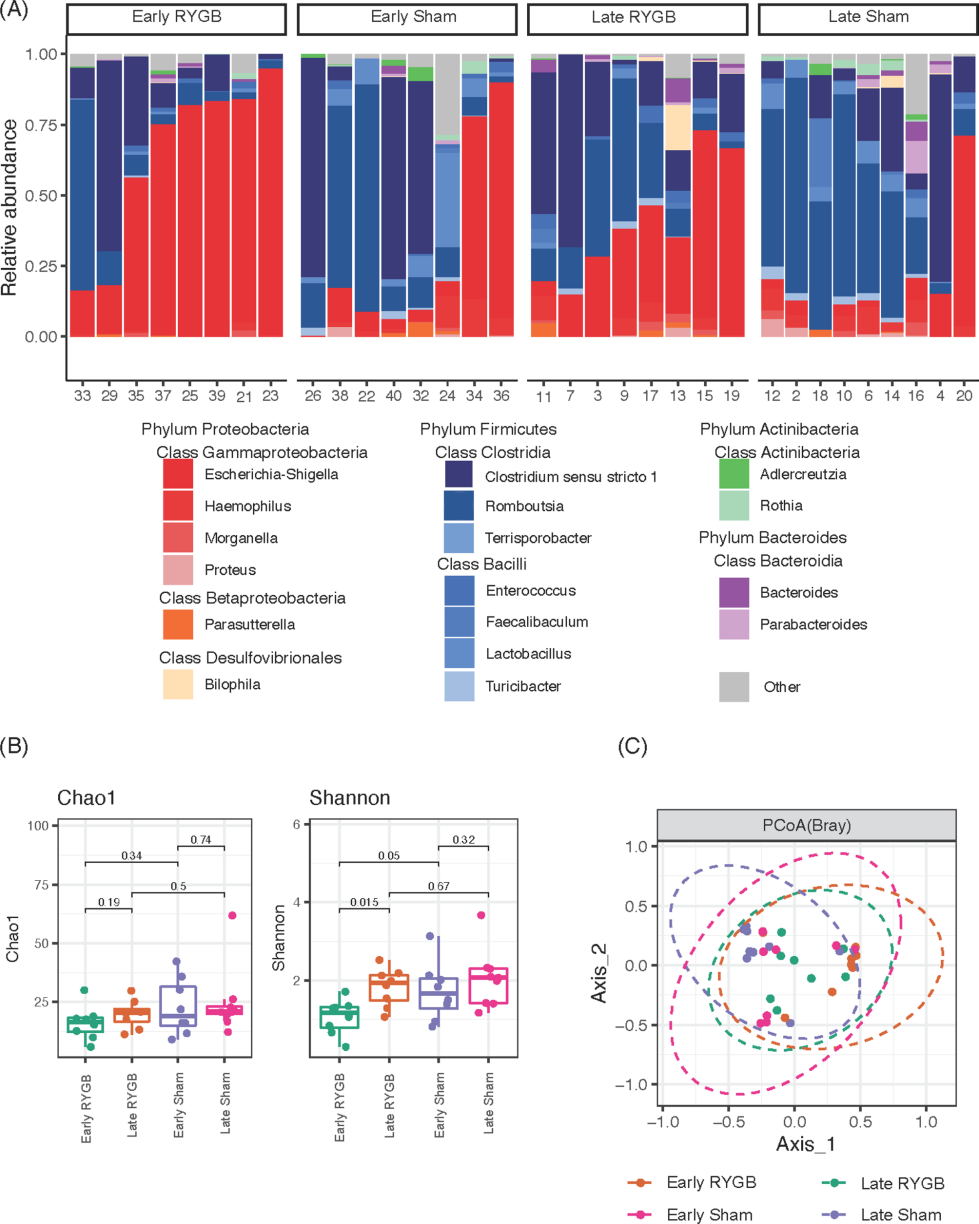
Differences in microbial abundance between Roux-en-Y gastric bypass and sham at early and late timepoints. (**A**) Taxonomic differences in microbial relative abundance between groups. (**B**) Between group differences in α diversity using the Chao1 and Shannon indices. (**C**) Between-group differences in β diversity using the Bray–Curtis dissimilarity index.

RYGB caused a significant shift in microbial composition, most prominently a large increase in relative abundances of *Escherichia–Shigella* species in both early and late cohorts (Fig. 4a). Alpha-diversity analysis revealed that early RYGB animals had significantly decreased evenness (Shannon index) when compared to early sham cohorts (*p* = 0.05). These differences were not present in the late cohorts due to restoration of diversity after RYGB (*p* = 0.015). There were no statistical differences in richness (Chao1 index) between any cohorts (Fig. 4b). Beta diversity approached statistical significance between the early RYGB cohort and early sham cohorts as determined by the Bray–Curtis dissimilarity index (*p* = 0.052). However, in the late groups, beta diversity was significant between RYGB and sham (*p* = 0.03, Fig. 4c). Average counts per sample was 49,328, ranging from 33,366 to 66,682 and rarefaction curves reached a plateau for all samples demonstrating that these reads included the vast majority of species (Supplementary Fig. 1).

### Microbial differences in abundances on univariate analysis

On the phylum level, there were higher Proteobacteria and lower Actinobacteriota in the early RYGB cohort compared to sham (Supplementary Fig. 2). On a genus level, early RYGB had higher abundances of *Escherichia–Shigella* but lower abundances of *Lactobacillus*, *Adlercreutzia*, and *Proteus* (Supplementary Fig. 3).

For the late cohorts, the higher abundance of Proteobacteria and lower Actinobacteriota became more significant with the addition of lower Firmicutes (Supplementary Fig. 4). On a genus level, the only significant differences were higher abundances of *Escherichia–Shigella* and lower abundances of *Lactobacillus* (Supplementary Fig. 5).

### RYGB results in decreases in total ileal bile acids and significant shifts in bile acid composition

**Figure 5:**
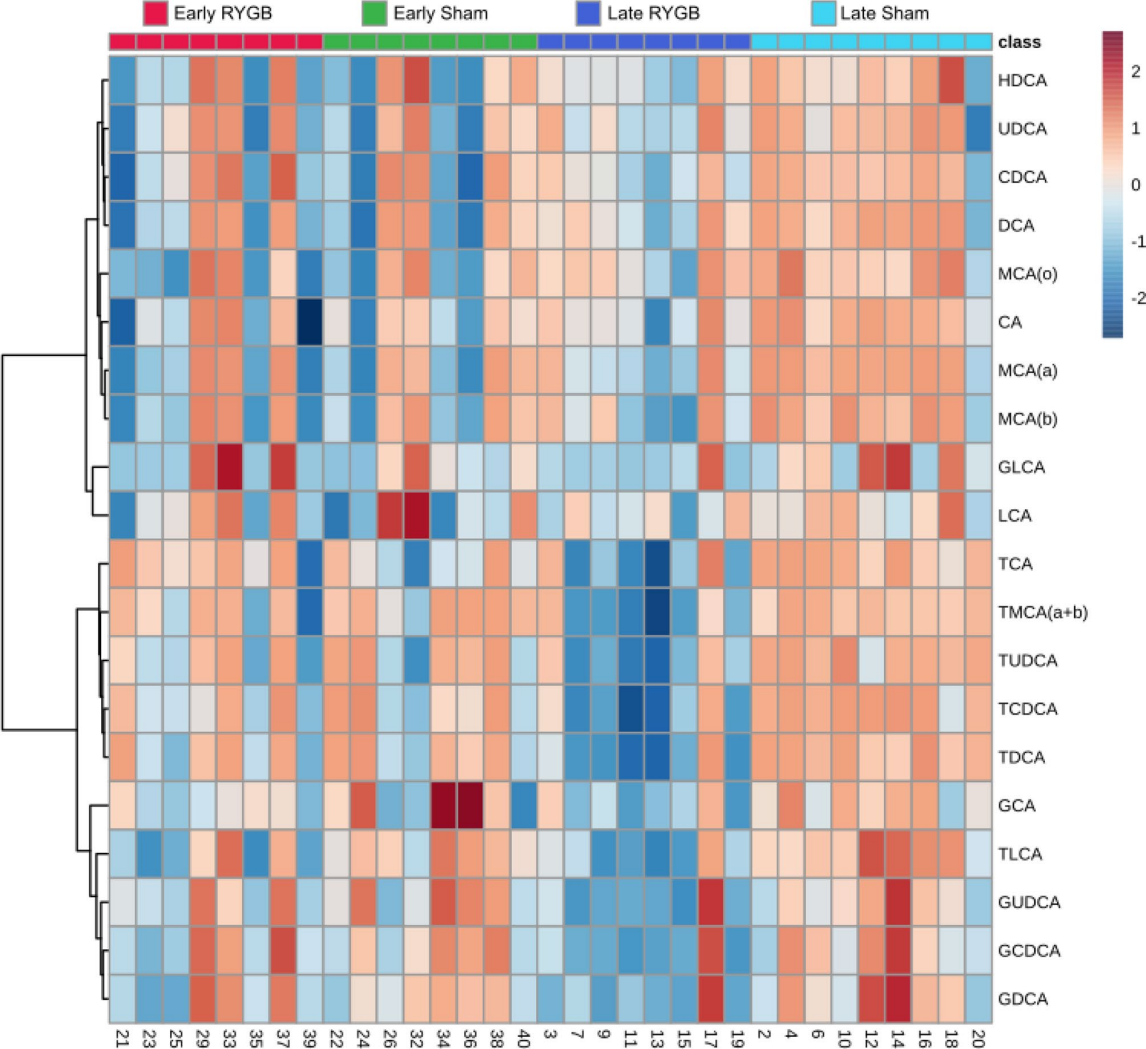
Heatmap of ileal bile acid concentrations after logarithmic transformation of data.

Among 20 ileal bile acids analyzed, there were no significant differences in the early cohorts. The late cohorts demonstrated significantly lower levels of seven primary bile acids and four secondary bile acids after RYGB (Fig. 5). Total bile acids were dramatically lower after RYGB compared to sham in the late groups (144.7 ± 205.7 vs. 408.8 ± 122.5 vs. µM, *p* = 0.0052). In the late groups, there were also higher cholic-acid-derived to chenodeoxycholic-acid-derived bile acid ratios after RYGB compared to sham demonstrating significant shifts in bile acid composition (2.87 ± 1.89 vs. 0.77 ± 0.15 µM, *p* = 0.0045).

### Shifts in microbial species were correlated with decreases in specific conjugated bile acids and with villi height

**Figure 6:**
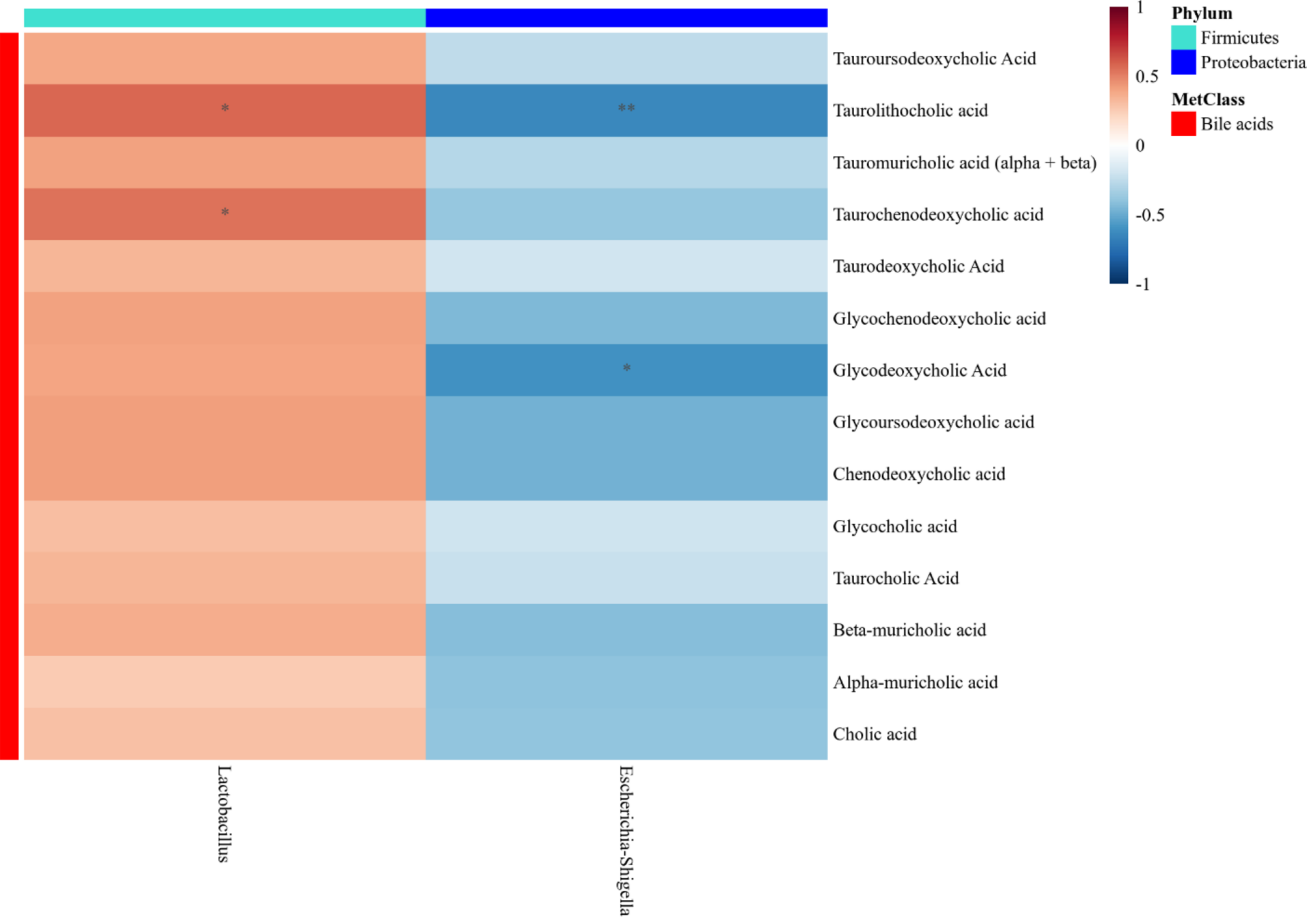
Heatmap of Spearman correlations of differential microbes and bile acids between late RYGB compared to late sham.

Pairwise correlation analysis between microbial and bile acid shifts did not reveal any significant correlations when comparing early sham to early RYGB. However, the late sham and late RYGB cohorts demonstrated positive correlations between *Lactobacillus* with taurolithocholic acid (R = 0.576, *p* = 0.016) and taurochenodeoxycholic acid (R = 0.546, *p* = 0.023) as well as negative correlations between *Escherichia–Shigella* and taurolithocholic acid (R =  − 0.642, *p* = 0.005) and glycodeoxycholic acid (R =  − 0.605, *p* = 0.01) (Fig. 6). Pairwise correlation between intestinal morphology, L-cell density and microbes revealed negative correlations between *Lactobacillus* and villi height (R =  − 0.485, *p* = 0.048).

## Discussion

To our knowledge, this is the first comprehensive analysis of metabolic changes assessed from the perspective of the terminal ileum following RYGB to evaluate the temporal relationships between bile acids, the gut microbiota, and L-cells in a diet-induced obesity rat model. Our study found that early ileal and metabolic changes were minimal after RYGB. However, late changes involved major shifts in bile acids and microbial composition which were associated with L-cells proliferation. In this discussion, we propose mechanisms to explain the changes observed in our study and present emerging evidence about the relationships between bile acids, the gut microbiota and L-cell proliferation.

Jejunal and ileal adaption occurring after RYGB was initially thought to occur due to compensatory mechanisms needed to overcome the decreased absorptive capacity of the alimentary limb^11,12^. However, an ileal interposition study suggested that these morphological processes are more complicated and may be mediated by increased nutrient and bile acid stimulation. Similarly, this study found intestinal hypertrophy and an increase in the total number of enteroendocrine cells^13^. Previous studies have demonstrated increases in L-cell quantity after RYGB with some demonstrating an increase in L-cell density^14,15^. Importantly, our study demonstrated no upregulation of GLP-1 associated gene expression suggesting that increased GLP-1 secretion is driven primarily by an increase in L-cell quantity rather than increased cellular production of GLP-1. Larraufie et al. similarly found no changes to the transcriptome or peptidome of L-cells after total gastrectomy with Roux-en-Y reconstruction^16^. Decreases in *Lactobacillus* were also associated with increased villi length. This was unexpected as studies in broiler chicken show that *Lactobacillus* supplementation increases villi height^17^. It is possible that the modified ecological or microbial environment after RYGB that causes increased villi height is inadvertently driving a loss in *Lactobacillus*.

A number of human RYGB studies have demonstrated an increased proportion of systemic circulating bile acids after RYGB and there are suggestions that these increases may correlate with remission of diabetes^18–20^. Reductions in luminal bile acid concentration are due to increased bile acid absorption after RYGB. Bhutta et al. demonstrated increased bile acid reabsorption in the proximal common jejunum but less reabsorption in the terminal ileum and colon. This may be related to the absence of bile in the Roux limb in conjunction with the absence of chyme in the biliopancreatic limb resulting in changes in the expression of genes related to bile acid absorption^21^. This is supported by an ileal interposition study which found increased bile acid reabsorption via apical sodium dependent bile acid transporter (ASBT) and an adaptive jejunization of the ileal segment due to its transposed location^22^. These findings are consistent with our study that found jejunization with lengthening of ileal villi as well as decreased concentrations of ileal bile acids.

There was a significant increase in Proteobacteria at a loss of Firmicutes in the late RYGB cohorts. One explanation is that phyla such as Firmicutes are more acid adaptive than Proteobacteria. RYGB leads to virtually absent acid secretion due to exclusion of the stomach and this leads to proportionally more alkaline pancreatic secretions flowing into the distal intestine^23,24^. This increased alkaline environment likely contributes to the shift towards Proteobacteria from Firmicutes that is occurring in our study.

The late RYGB cohort had an increase in *Escherichia–Shigella* and a decrease in *Lactobacillus*. This is consistent with multiple studies that demonstrate an increase in *Escherichia–Shigella* and decrease in *Lactobacillus* after RYGB^7,25,26^. Increases in *Escherichia–Shigella* correlated to decreases in taurolithocholic acid and glycodeoxycholic acid while decreases in *Lactobacillus* correlated to decreases in taurolithocholic acid and taurochenodeoxycholic acid. One mechanism in which *Lactobacillus* decreases bile acids is via its bile salt hydrolase (BSH) activity in the small bowel. Decreases in the abundance of *Lactobacillus* would lead to decreases in BSH activity, lowering levels of bile acid deconjugation, increasing the amount of bile acid reuptake, which may result in lower intraluminal bile acid levels^27^. Additionally, species such as *E. coli* exhibit bile salt oxidation and epimerization via hydroxysteroid dehydrogenase. Epimerization is a stereochemical change from an α to β configuration, with the formation of stable oxo-bile salt intermediates. Modified bile salts are typically reabsorbed and this contributes to reduced luminal concentrations of bile acids^28^. These processes potentially connect shifts in ileal bile acids to the microbial shifts that occur after RYGB through organism such as *Escherichia–Shigella* and *Lactobacillus*.

*Lactobacillus* was consistently decreased in both the early RYGB cohort and the late RYGB cohort. *Lactobacillus* is a gram-positive, aerotolerant anaerobic bacterial species often used as a probiotic. When used as a probiotic, there is emerging evidence that it has positive effects on glucose metabolism^29,30^. However, the role of *Lactobacillus* after RYGB is incongruent with these studies as its abundance after RYGB was found to be lower in our study as well as studies by Furet et al. and Kong et al.^25,26^. This decrease is thought to occur due to intraluminal increases in pH which tend to demote acidophilic genera such as *Lactobacillus*. *Lactobacillus* is also particularly adaptable to bile acids environments and reductions in luminal bile acids after RYGB may also contribute to decreases in its abundance^31^.

One of the potential mechanisms for L-cell proliferation is through bile acid signaling. Bile acids have demonstrated the ability to directly cause intestinal L-cell differentiation and increases in density. A study by Lund et al. found that both lithocholic acids and synthetic GPBAR1 agonists increased L-cell density and GLP-1 secretory capacity^32^. Bile acids also have direct effects on L cells through the FXR and TGR5 receptors^33–35^. For example, TaMCA and TbMCA were demonstrated in two studies to inactivate intestinal FXR and prevent diet-induced obesity and improve glucose metabolism^36,37^. In our study, we found decreases in 11 bile acids in the late RYGB cohort and these shifts may contribute to signaling towards L-cell proliferation. Future studies directed at these bile acids may identify their effect on L-cells.

There is also emerging evidence that the gut microbiota within the ileum have rapid and pronounced effects on L-cells and GLP-1. Arora et al. studied germ-free mice and found that the recolonization of the ileal microbiota downregulated the production of GLP-1 through genes related to vesicular localization^3^. This occurred rapidly within one day of recolonization. Yoon et al. found that *Akkermansia muciniphila* secretes a protein that specifically induces the release of GLP-1 from intestinal L-cells^38^. Other studies have found that colonic *A. muciniphili* increases after RYGB^39,40^. However, high-fat diets have been demonstrated to significantly reduce the abundance of this bacteria and this could explain why our samples yielded sparse abundances of *A. municiphila* in all cohorts^41,42^. Our study primarily found increases in *Escherichia–Shigella* and decreases in *Lactobacillus* after RYGB and future studies directed at these species may identify if these species have effects on L-cells.

### Limitations

Our study is the first to perform a comprehensive assessment of the rat ileum after RYGB to determine factors that may contribute to changes in L-cells. However, our study was not specifically designed to evaluate the underlying mechanisms responsible for the proliferation of L-cells and serves primarily as hypothesis generating. This study also did not demonstrate early improvement in glucose tolerance. This may be because our study used an obesity model and not a diabetic rat model which may have attenuated the early metabolic effects of RYGB. Importantly, rats were fed ad-libitum prior to plasma GLP-1 testing and this may have contributed to the lack of significant difference in GLP-1 between groups as the timing of postprandial GLP-1 can affect results. Another important limitation is that bile acid physiology has major differences between rats and humans.

In conclusion, there were no early changes to L-cells, bile acids, or glucose homeostasis after RYGB. However, RYGB caused a late and substantial increase in L-cell quantity with associated shifts in ileal bile acids which correlated to shifts in *Escherichia–Shigella* and *Lactobacillus*. This proliferation of L-cells contributed to increased GLP-1 secretion and improved glucose homeostasis. This study demonstrates that both foregut and hindgut theories are overly simplified and that the intestinal changes that contribute to L-cell proliferation are multimodal and complex. L-cells appear to be key players in the regulatory mechanisms associated with RYGB and more research is needed to elucidate the complex interplay between L-cells, bile acids and the gut microbiota.

## Methods

### Study design

All animal experiments complied with the ARRIVE guidelines and the study was approved by the Animal Research Ethics Board at the University of Alberta (AUP00003000). All methods were performed in accordance with the relevant guidelines and regulations. Thirty-six male Wistar rats were randomly assigned to four cohorts based on procedure and planned date of euthanasia: 2-week RYGB, 14-week RYGB, 2-week sham, or 14-week sham. Male rats were chosen to avoid estrous cycles of female rats which may affect hormone outcomes in the 2-week cohorts. The rats were doubly housed until 6 weeks of age after which they were separated into single cages to avoid cage effects biasing microbial analysis. Sterile HFD (Bio-Serv S3282, 60% calories from fat) was introduced at 6 weeks of age and continued throughout the experiment, except during the perioperative period. Body weight was monitored weekly. RYGB or sham surgery were performed at 16 weeks of age. Importantly, this study was a model of severe obesity and not of diabetes. Study flowchart is detailed in Supplementary Fig. 6.

Rats were euthanized at 2 and 14 weeks after surgery to evaluate early and late post-RYGB L-cell and enteroendocrine changes. Euthanasia occurred with deep isoflurane anesthesia via cardiac puncture. The 2-week time point was chosen to evaluate early changes to allow for a 1-week washout from postoperative liquid diet and to assess for metabolic changes while on HFD. The 14-week timepoint was chosen to allow for evaluation of late RYGB-adaptive L-cell changes. One week prior to euthanasia, intraperitoneal glucose tolerance testing (IPGTT) was performed. Rats were then euthanized with collection of blood for postprandial gut hormones, ileal tissue and ileal enteric contents. Ileal tissue underwent immunofluorescence staining to quantify the number of L and K cells. Intestinal morphology quantification was conducted with direct microscopy. Reverse transcription polymerase chain reaction (RT-PCR) was performed on ileal tissue for gene expression of GLP-1 and gastric inhibitory polypeptide (GIP) relevant genes. Ileal enteric contents underwent 16S rRNA sequencing for microbial composition and liquid chromatography-mass spectrometry for bile acid analysis.

### Study objectives

The primary objective of this study was to determine early and late changes to L-cell quantity and GLP-1 gene expression of ileal L-cells between RYGB and sham surgery, defined as 2 weeks and 14 weeks, respectively.

Secondary objectives included determining changes in villi morphology, bile acid composition, and microbial composition within the ileum and identifying pathways in which these changes affect L-cell gene expression and quantity. Additionally, serum GLP-1 and glucose tolerance were compared between groups to determine the effects of RYGB on glucose metabolism.

### Sample size calculation

Sample size calculations were designed to ensure GLP-1 changes induced by surgery would be adequately captured. In prior literature, rats had significantly increased GLP-1 after RYGB (25 vs. 75 pmol/L, σ = 30)^43^. Powering to detect a 25% GLP-1 difference between cohorts, with an alpha of 0.05 and a beta of 0.80, would require 8 rats per arm. Also accounting for a mortality of ~ 10%, this would require 9 rats per arm for a total of 36 rats.

### Surgical procedure

After an overnight fast, RYGB (Supplementary Fig. 7) or sham surgery were performed based on previously published protocols^44^. In summary, anesthesia was induced using isoflurane and rats were given subcutaneous buprenorphine sustained release (1 mg/kg). A midline incision was made sharply, and the stomach was mobilized by dividing the gastric attachments using a combination of electrocautery and ligation with 6-0 polypropylene. A window was made in the gastrohepatic ligament superior to the left gastric artery to allow for stapling. The ligament of Treitz was located and the jejunum was divided 7 cm distally. The stomach was divided with a 45 mm laparoscopic linear stapler (Ethicon, ETS45) with 3.5 mm blue load staplers. Hemostasis at the staple line was achieved with pressure and suture ligation. A second stapler was deployed to resect the gastric fundus (forestomach) to prevent retained food within a large pouch. A gastrotomy was created in the distal pouch. A circular gastrojejunostomy was created with 6-0 polypropylene (continuous on anterior side, interrupted on posterior). A leak check was performed by gently compressing enteric contents through the anastomosis. The jejunum was then measured 20 cm distally and an enterotomy was created. An end to side jejunojejunostomy was then performed using a similar technique to the gastrojejunostomy. The fascia was closed using 3-0 polyglactin and skin was closed with 2-0 silk.

The sham procedure was performed similarly except a gastrotomy was made in the distal anterior stomach and closed with 6-0 polypropylene. A jejunotomy was made 7 cm distal to ligament of Treitz and closed with 6-0 polypropylene.

Postoperatively, the rats were provided water and electrolyte replacement solution (Hydralyte) for 72 h but given twice daily subcutaneous D5NS solution. On postoperative day 3, the rats were progressed to a liquid diet (Bio-Serv, F1259) and then resumed on high-fat diet on day 5.

### Intraperitoneal glucose tolerance test

An IPGTT was performed 1 week prior to euthanasia. After a 16-h fast, blood glucose was measured from the lateral saphenous vein of unrestrained rats using a glucometer at baseline and following intraperitoneal dextrose injection at a dose of 2 g/kg of body weight. Post-injection glucose measurements occurred at 15, 30, 60, and 120 min.

### Serum enzyme-linked immunosorbent assay (ELISA) analysis

Rats were fasted overnight for 12 h prior to euthanasia and then given ad-lib access to high-fat diet for 3 h. Blood was collected by cardiac puncture immediately prior to euthanasia for gut hormone testing. A protease inhibitor cocktail (Thermal Fisher) was added, and serum was stored in a freezer at − 80 °C. Serum was analyzed for total GLP-1 (Millipore, EZGLP1T-36K) and total GIP (Millipore, EZRMGIP-55K) using standard ELISA.

### Isolation of intestinal cells, brightfield microscopy, and immunofluorescence

After rats were anesthetized, a 10-cm segment of ileum proximal to the cecum was removed. Intestinal tissue was opened longitudinally and rinsed with phosphate-buffered saline. Separate portions were flash frozen in a guanidinium thiocyanate solution (Thermo Fisher, Trizol) and cryopreserved in neutral buffered formalin (10% vol:vol) with sucrose^45^. These were fixed using a standard alcohol, xylene and paraffin process. Embedded tissue was cut to 16 µm and mounted on a slide.

Ileal morphology was examined using brightfield microscopy (Zeiss, Observer Z1) at 10× magnification. Twenty random villi and crypts were measured for villi height, villi width, crypt width, crypt depth and epithelial thickness. Only complete and vertically oriented villi and crypts were measured (Supplementary Fig. 8).

Immunofluorescence staining was performed as per published protocols^46^ using Polyclonal GIP (Thermo Fisher, PA5-76867, 1:800) and Anti-GLP1 (Abcam, ab26278, 1:800) primary antibodies with Alexa Fluor 488 (Thermo Fisher) and Alexa Fluor 647 (Thermo Fisher) secondary antibodies. This allowed visualization of L-, K- and L-cells which co-express GLP-1 and GIP. Nuclei staining was performed using diamidino-2-phenylindole (DAPI) solution (Thermo Fisher).

Forty random confocal images of epithelium were taken at 40× magnification using the WaveFX Confocal microscope. Images were manually counted for L-cells and K-cells. Cell density was calculated based on cells/mm^2^. Supplementary Figure 9 is a representative image of resultant immunofluorescence staining.

### Quantitative RT-PCR

RNA was extracted from ileal intestinal tissue using the TRIzol® Plus RNA Purification Kit (Thermo Fisher, 12183555). Reverse transcription was performed with the High-Capacity cDNA Reverse Transcription Kit (Thermo Fisher, 4368814). Quantitative PCR was performed with the TaqMan Gene Expression Master Mix and the following mRNA sequences:

- gcg (Proglucagon mRNA) for GLP-1 expression.
- PC1/3 (Prohormone convertase 1/3 mRNA)—mediates posttranslational processing of proglucagon.
- gip (Pro.GIP mRNA) for GIP expression.
- Normalized to CgA (Chromogranin A) gene, specific for enteroendocrine cells.
- Corrected against RPL32 (ribosomal protein 32)—housekeeping gene.

### Ileal microbial analysis

The microbial community compositions of ileal enteric contents were assessed using 16S rRNA gene sequencing. DNA was extracted from ileal homogenates combining enzymatic and mechanical cell lysis with the QIAamp DNA Stool Mini Kit (Qiagen, USA). Enteric microbiota composition was characterized by 16S rRNA tag sequencing using the MiSeq Illumina technology (pair-end), targeting the V3–V5 regions. This analysis was performed by Genome Quebec (Montreal, Canada).

Demultiplexed FASTQ sequences were quality filtered, trimmed, dereplicated, and filtered for chimeric sequences using pair-ended DADA2 resulting in exact sequence variant (feature) tables^47^. This methodology was chosen because amplicon sequence variant analysis demonstrates higher resolution and accuracy compared to the construction of operational taxonomy units^48^. The feature table was imported into R 3.6.1 to analyze for α-diversity (Shannon), β-diversity (wunifrac) and were performed using a function of the phyloseq v1.28.0 package^49^. Ordination plots for β-diversity metrics were generated by non-parametric multidimensional scaling ordination in R.

### Ileal bile acid analysis

Bile acid analysis was performed for quantification of 20 rodent specific bile acids in rat ileal fecal matter using the AbsoluteIDQ bile acids kit (Biocrates) and liquid chromatography-mass spectrometry. This was performed at The Metabolomics Innovation Centre (Edmonton, Canada) and includes quantification of unconjugated, taurine- and glycine-conjugated bile acids.

### Statistical analysis

Descriptive categorical data were expressed as percentages and continuous data were expressed as mean ± standard deviation (SD). Baseline differences between groups were evaluated by univariate analyses using Fisher’s exact test for categorical data and independent sample t-test for continuous data. Multiple comparisons were adjusted using the Benjamini–Hochberg method to correct the false discovery rate. Error bars on figures represent standard error of the means. Analyses were conducted using STATA 15 (StataCorp 2017; College Station, TX). Figures were designed using Prism 9.0.0 (GraphPad Software, https://www.graphpad.com/, San Diego, CA). Statistical significance was defined using two-tailed tests with a *p* value < 0.05.

Integrated microbial and bile acid analysis was performed using the M^2^IA platform^50^. Microbial abundance counts were normalized by percentages. Differential bile acids and microbial relative abundance between groups were selected with univariate analysis using the Student t-test for bile acids and the Mann–Whitney U test for microbial abundance. Spearman’s correlation coefficients were calculated between differential bile acids and microbes using a pairwise correlation analysis method with significance defined as *p* < 0.05 and R > 0.3 or <  − 0.3. Spearman’s correlation was also performed between intestinal morphology, L-cell density and microbes.

## Supplementary Information

Supplementary Information.
