# Supplementary material for: Ileal microbial shifts after Roux-en-Y gastric bypass orchestrate changes in glucose metabolism through modulation of bile acids and L-cell adaptation
Source: Sci Rep. 2021 Dec 10;11:23813. doi: 10.1038/s41598-021-03396-4 (PMC8664817; doi:10.1038/s41598-021-03396-4)
Supplement: Supplementary file 1 — Supplementary Information. [file 41598_2021_3396_MOESM1_ESM.docx]

## Supplementary Figures


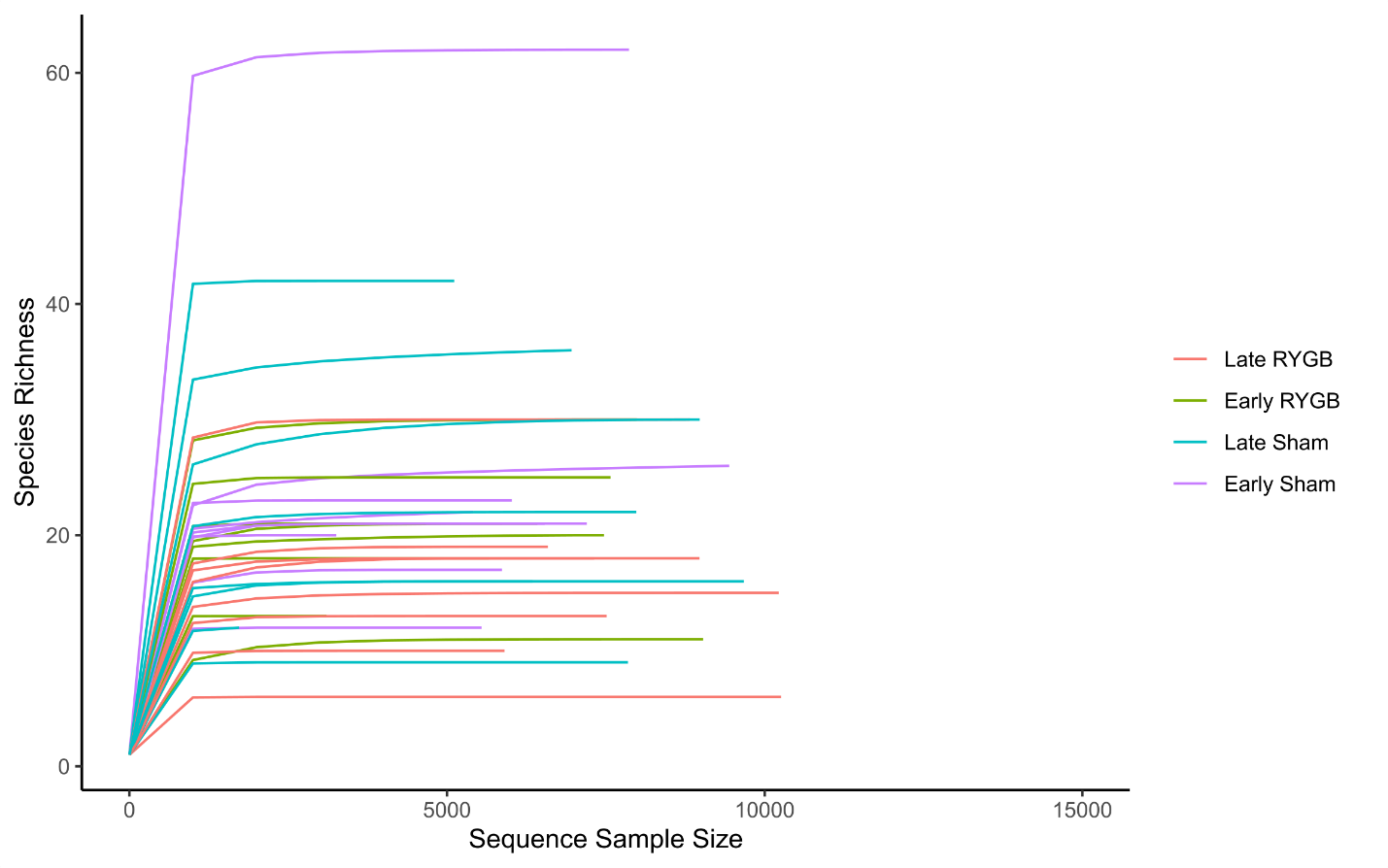


Supplementary Figure 1. Rarefaction curves. RYGB; Roux-en-Y gastric bypass.


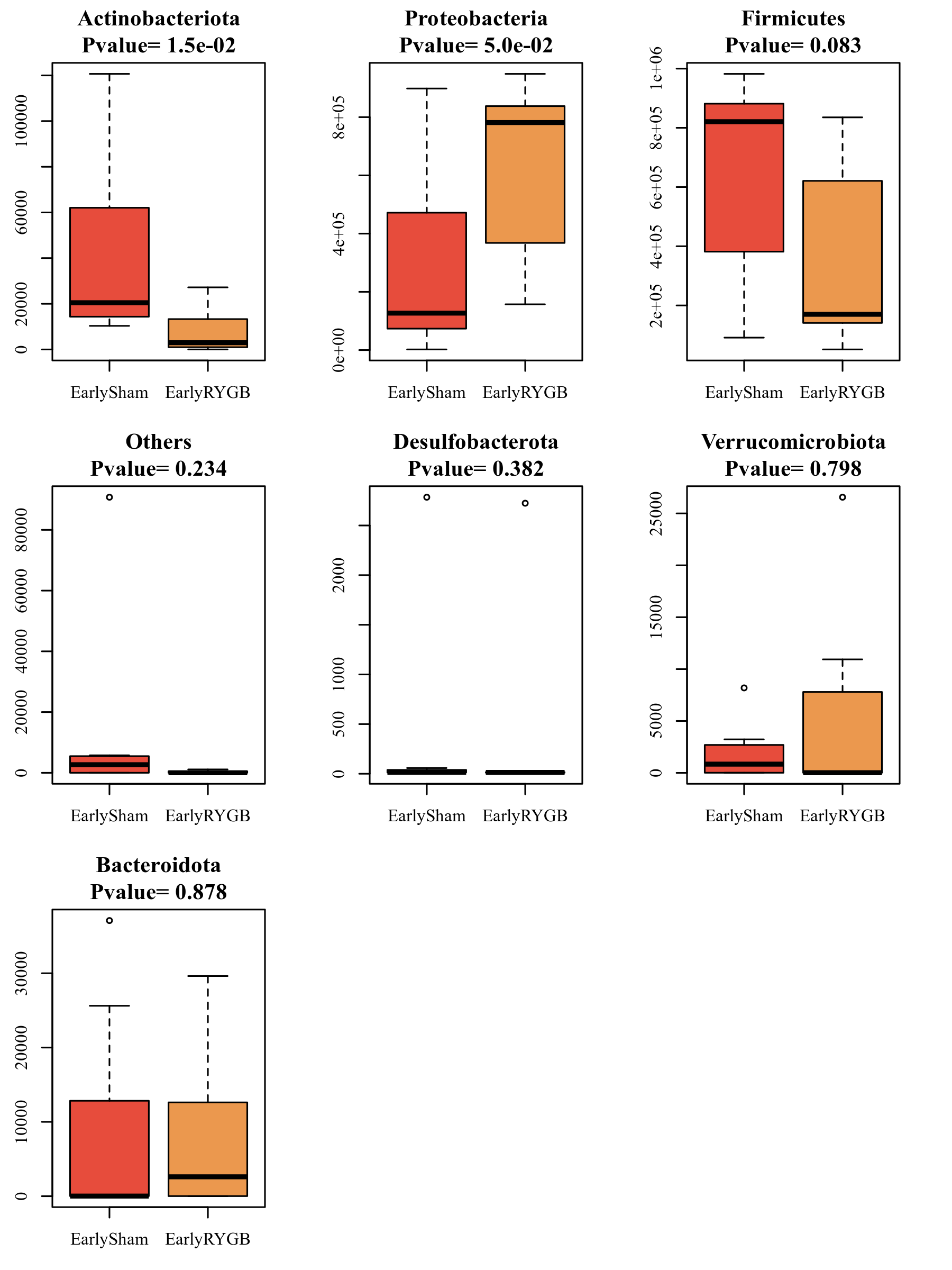


Supplementary Figure 2. Differential microbial relative abundance on univariate analysis at the phylum level between early sham cohorts and early Roux-en-Y gastric bypass cohorts


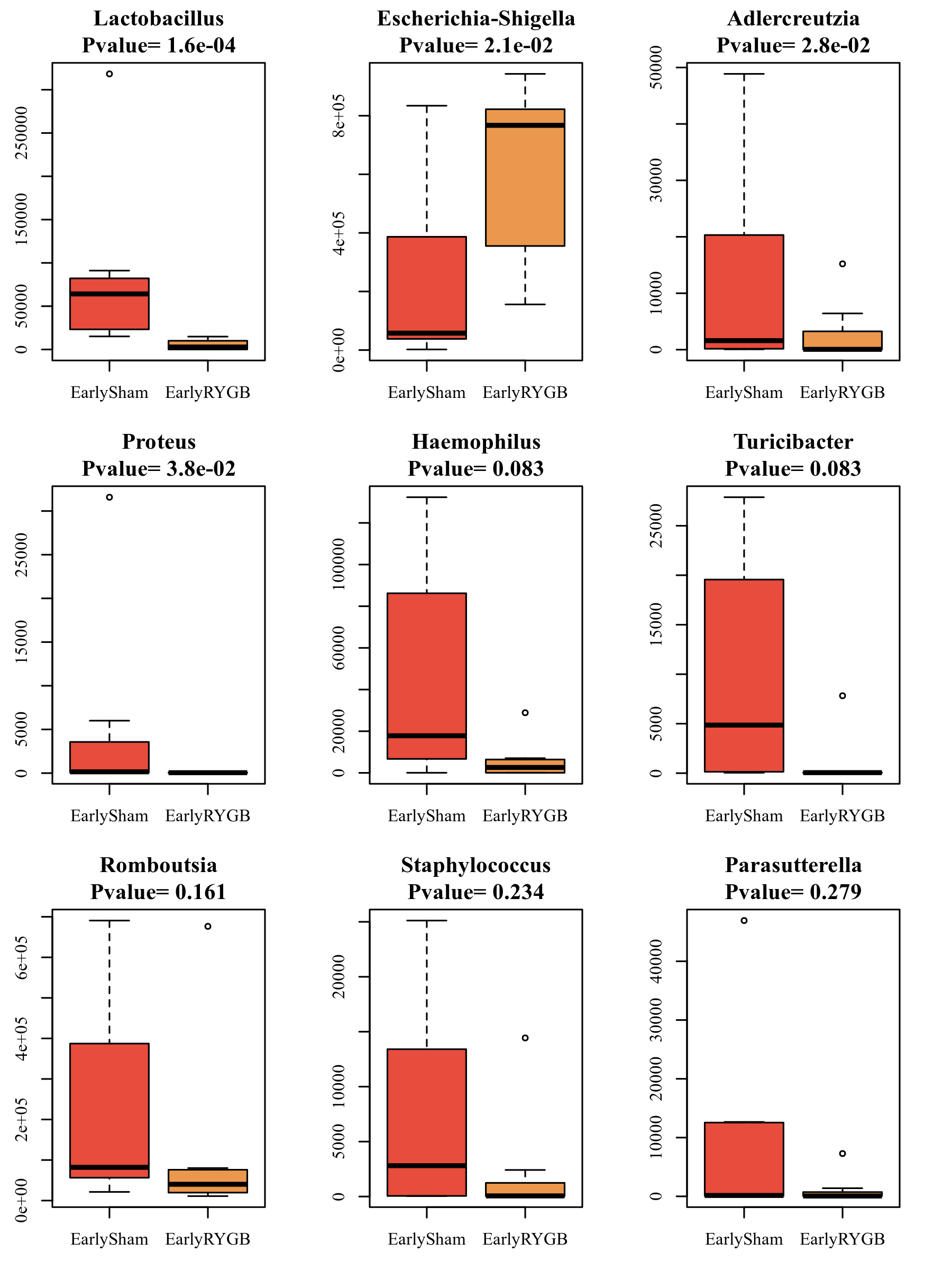


Supplementary Figure 3. Differential microbial relative abundance on univariate analysis at the genus level between early sham and early Roux-en-Y gastric bypass cohorts


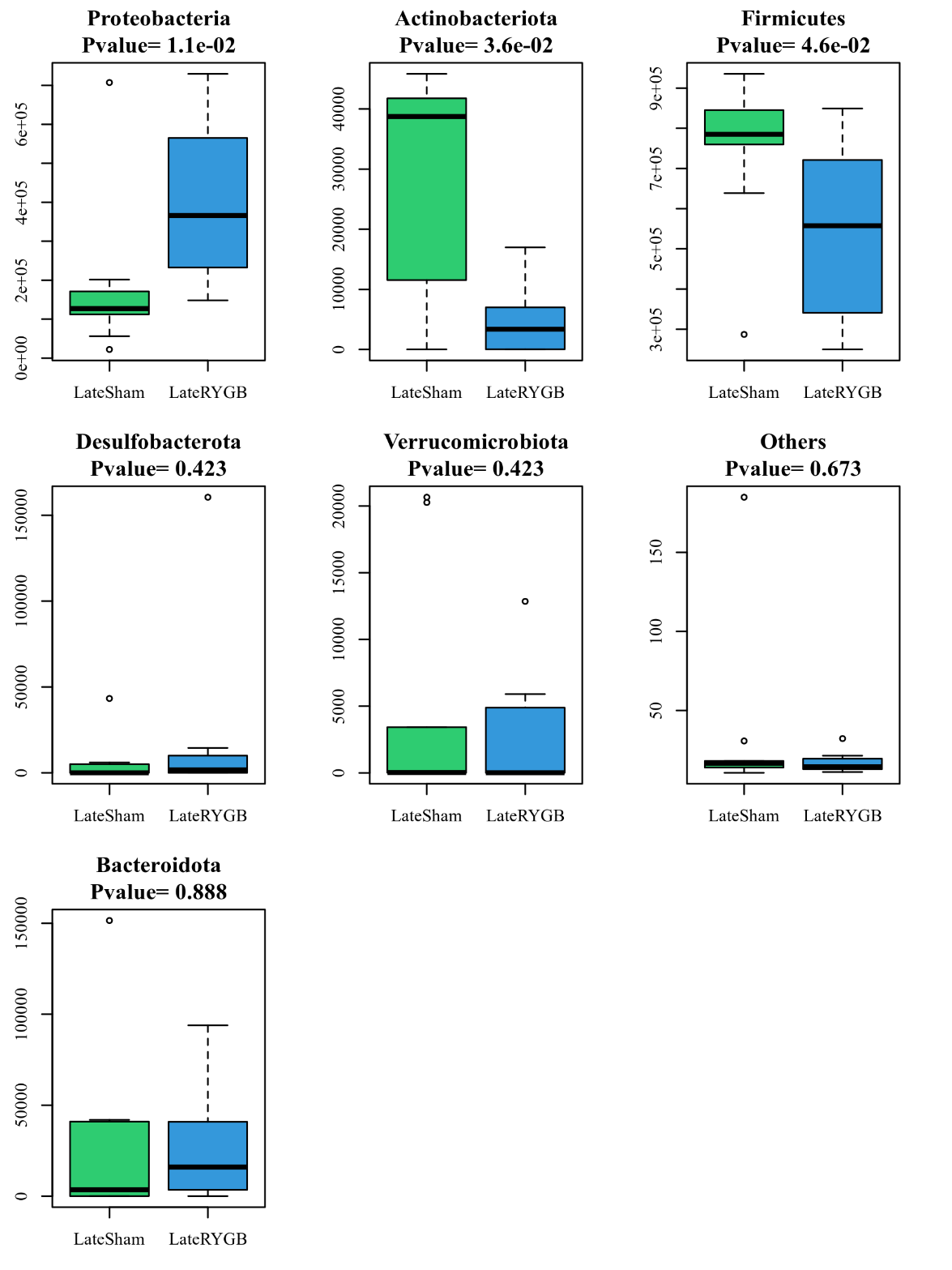


Supplementary Figure 4. Differential microbial relative abundance on univariate analysis at the phylum level between late sham and late Roux-en-Y gastric bypass cohorts


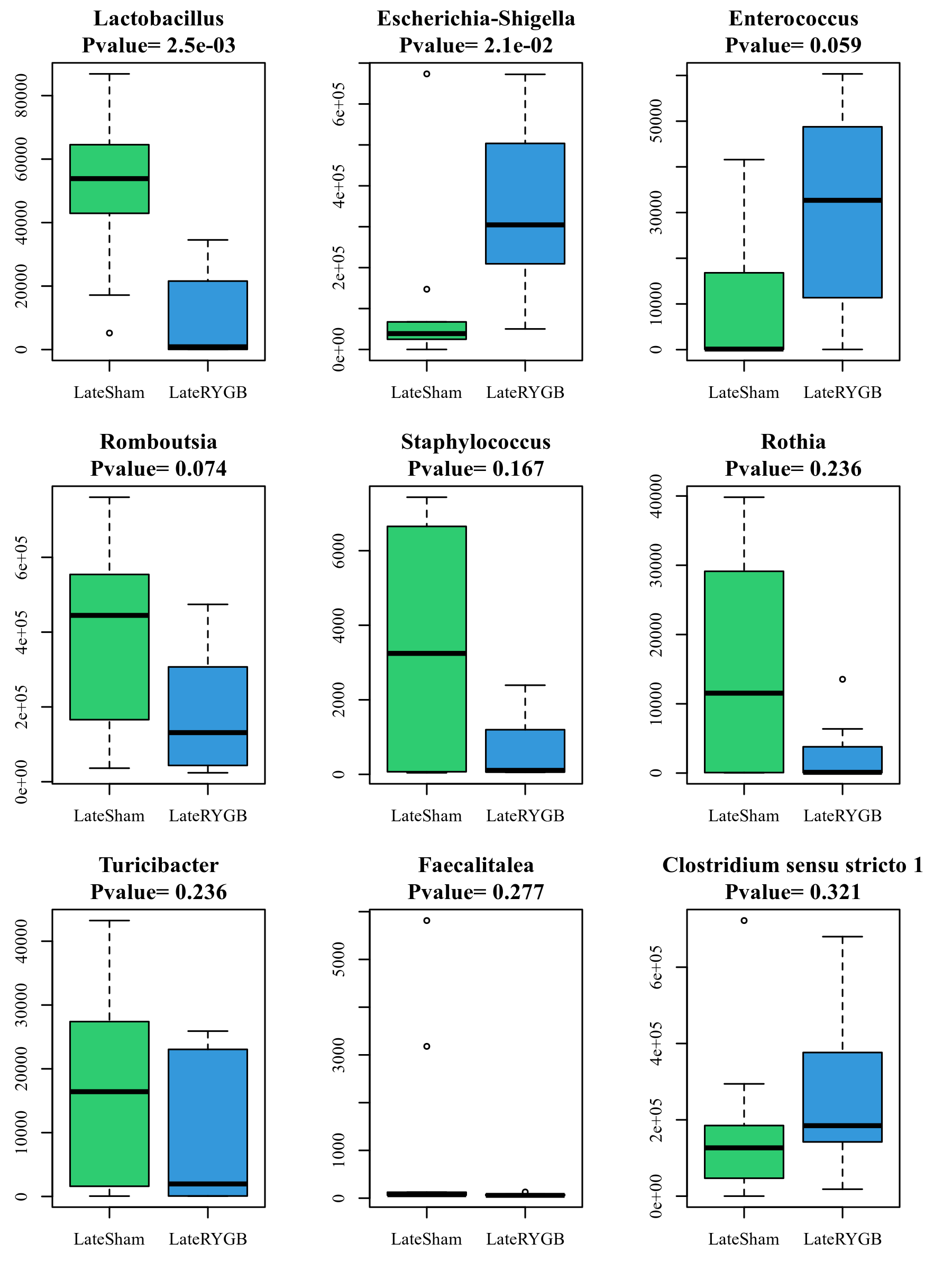


Supplementary Figure 5. Differential microbial relative abundance on univariate analysis at the genus level between late sham and late Roux-en-Y gastric bypass cohorts

Supplementary Figure 6. Study flowchart and timeline. RYGB, Roux-en-Y gastric bypass.


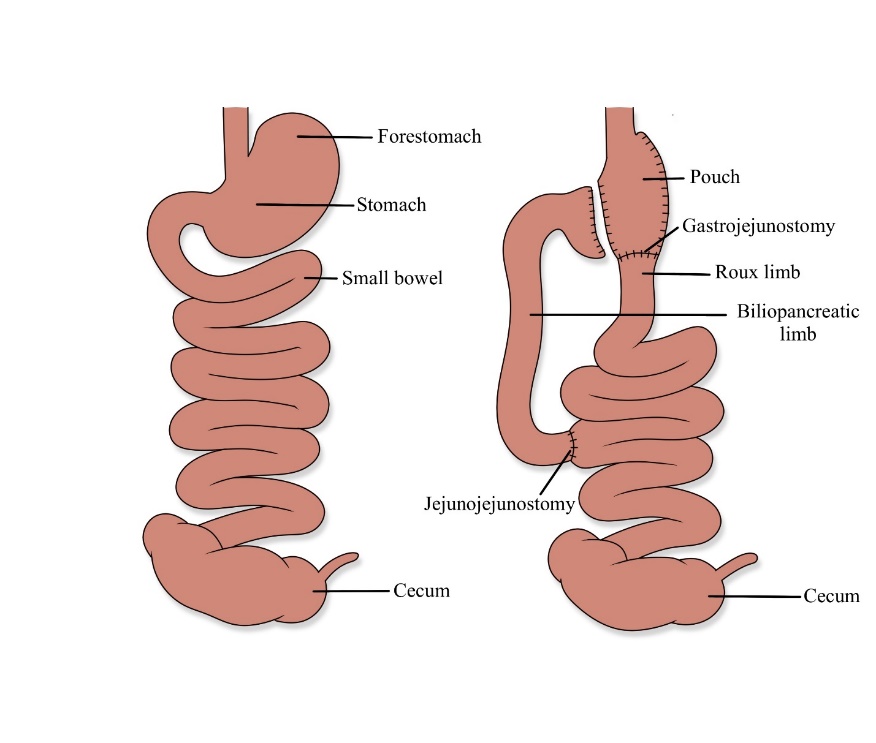

Supplementary Figure 7. Roux-en-Y gastric bypass anatomy in the rat. Image drawn by Michelle Tran.


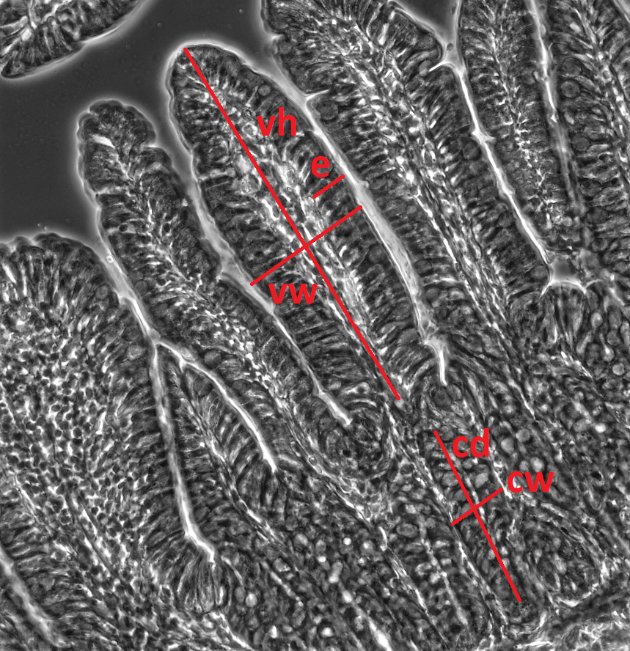


Supplementary Figure 8. Cross section of the ileum showing the measured parameters: villus height (vh), villus width (vw), crypt depth (cd) and crypt width (cw)


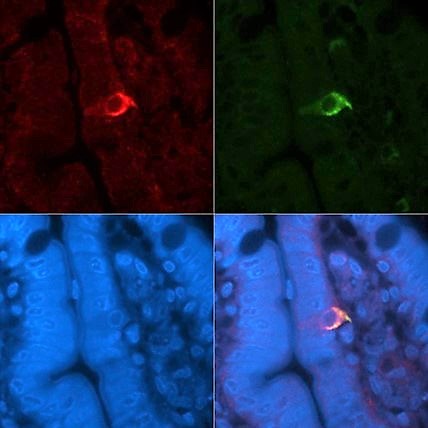


Supplementary Figure 9. Immunofluorescence staining of an LK cell. Red is GIP stain, green is GLP-1 stain and blue is DAPI stain for nuclei
